# Spatial and Temporal Distribution of Pollution Based on Magnetic Analysis of Soil and Atmospheric Dustfall in Baiyin City, Northwestern China

**DOI:** 10.3390/ijerph18041681

**Published:** 2021-02-09

**Authors:** Bo Wang, Xiaochen Zhang, Yuanhao Zhao, Mei Zhang, Jia Jia

**Affiliations:** College of Geography and Environmental Sciences, Zhejiang Normal University, Jinhua 321004, China; zhangxiaochen@zjnu.edu.cn (X.Z.); zhaoyuanhao@zjnu.edu.cn (Y.Z.); zhangmei@zjnu.edu.cn (M.Z.); jiaj@zjnu.edu.cn (J.J.)

**Keywords:** magnetic properties, topsoil, atmospheric dustfall, mining city, pollution

## Abstract

The characteristics of spatial-temporal distribution and sources for multiple environmental carriers (surface soil, soil profiles, atmospheric dustfall) from the typical industrial city of Baiyin in Northwestern China were studied by means of environmental magnetism. This study aims to contribute to the potential application of magnetic measurements in the case of multiple environmental carriers, for the evaluation and differentiation of urban pollution sources. Results show that background magnetic susceptibility of soil is 37 × 10^−8^ m^3^ kg^−1^, and that magnetite and hematite carry the magnetic properties. However, magnetic properties of urban soil and atmospheric dustfall are dominated by PSD magnetite. Magnetite content in soil samples is anomalously high surrounding metallurgical plant and slag dump (major industry district), of moderate value in the center of the city (major commercial district), and of low value in the west of city (Baiyin new zone). Vertical distribution of magnetite content in soil profile of waste land suggests that the pollutants are mostly enriched in the top 0–2 cm soil layers, while planting of crops near the industrial area may accelerate the transfer of contaminants deeper in the soil (2–30 cm); accordingly, reducing detrimental soil tillage practices can alleviate the vertical migration of pollution. Measurements of magnetic variations of atmospheric dustfall indicate that industrial emissions by factory chimneys and blowing dust from slag heap and mineral transport control the magnetic properties of dust, with slag heaps being the main pollution source since 2014. Governance of slag pollution is a primary task in resource-exhausted urban contexts. The combination of several magnetic parameters arising from multiple environmental carriers, such as soil and atmospheric dustfall, can provide comprehensive spatio-temporal information on environmental pollution.

## 1. Introduction

Urban pollution due to global expansion of industrialization and urbanization is a worldwide issue and is seriously harmful to human health and urban ecosystems. Hazardous pollutants lurk in urban soils, sediments, dusts or leaves and are derived from different kinds of human activities, e.g., metallurgical processes, fossil fuel combustion, motor vehicles and bare soils, jeopardizing human health through resuspension and inhalation. Numerous studies have linked the synergistic effects of different urban contaminant (e.g., PM_10_, PM_2.5_) to a high number of cardiovascular, respiratory disease and cancer deaths [1,2,3,4]. Therefore, it is necessary to rapidly estimate the concentration of hazardous dusts and their emission sources.

Environmental magnetic monitoring may provide a robust and cost-effective means to achieve high density mapping and monitoring of heavy metals in soils, sediments, street dust and atmospheric dustfall, tracing their sources and reconstructing pollution history [5,6,7,8]. This method is based on the close link between certain major hazardous pollutants (e.g., heavy metals) and the ferrimagnetic fraction of particulate matter, thus providing a qualitative, and semi-quantitative estimate of the level of environmental pollution [9,10,11,12,13,14,15,16]. 

Generally, magnetic analysis is applied to environmental carriers, such as soil, road dust, atmospheric dustfall and leaves of vegetation [6,14,17,18]. Topsoils are suitable for monitoring the spatial distribution in urban, road and industrial areas, and reflect the accumulation of anthropogenic dusts over longer periods, while roadside dusts provide contamination information over a relatively short period. Soil profiles reflect the transport process of pollutants, and atmospheric dustfall reveals the temporal dynamics of pollution over a period. These carriers are indicative of pollution from natural sources, such as bare soils, and from anthropogenic processes, whose relative contributions may vary significantly both spatially and temporally. Unfortunately, little information is available regarding the magnetic properties of different environmental carriers [10,19,20]. Thus, the major aim of this paper is to determine the magnetic ‘fingerprint’ of different environmental carriers from one city, and to study how different environmental carriers can reflect urban pollution distribution over time and space. Thus, we selected the typical mining city of Baiyin to analyze the magnetic characteristics of dust. In so doing, we explored magnetic parameters for surface soil, soil profile and atmospheric dustfall to reveal the lateral, vertical, and temporal distribution of pollutants in Baiyin city and to identify the contribution of different pollution sources.

## 2. Methods

### 2.1. Sampling Methods

**Figure 1:**
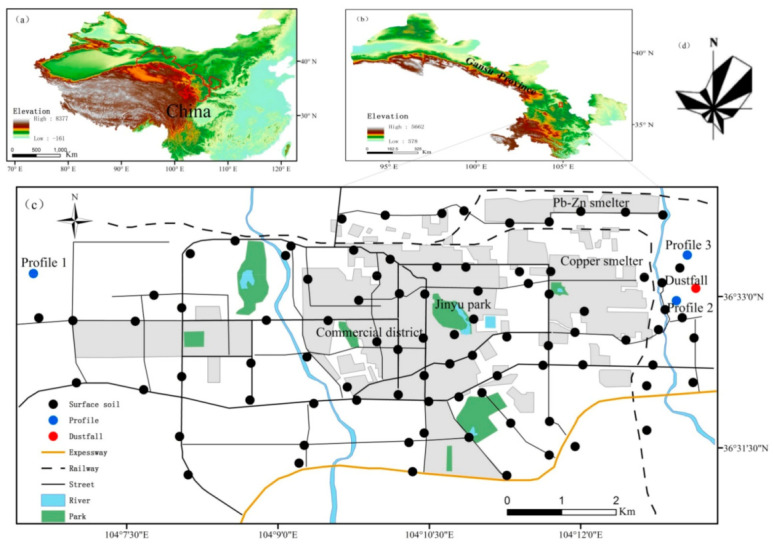
(**a**) Location of Gansu province in China; (**b**) Location of Baiyin city in Gansu province; (**c**) Sketch map of the study area; (**d**) Wind direction rose map of Baiyin.

Baiyin city is located close to the northwest margin of the Chinese Loess Plateau (Figure 1a), and has a semi-arid climate (mean annual precipitation approximates 250 mm). The surrounding landscape is characterized by gray calcareous soil and is covered by true temperate steppe vegetation. In the urban area, the soil derived from loess material and yields a sandy loam texture, with low organic matter content (mean 1.85%) [21]. Baiyin is an important non-ferrous metals mining and smelting city in China. Several large mining and industrial companies, occupying a vast area, were established to extract lead-zinc, copper and polymetallic ores during the 1970’s. Nowadays, Baiyin is declining as the resources are becoming exhausted.

Topsoil samples were collected 2 to 3 m from the edge of roads, and four sampling point within 10 m were combined to form one composite sample for analysis. A total of 87 samples (S1–S87) was collected. The sampling locations are shown in Figure 1. Furthermore, three soil profiles were sampled in a suburb near Baiyin city. Profile 1 was obtained from a wasteland located in the western part of Baiyin city, Profile 2 was obtained from a harvested agricultural field and Profile 3 from a wasteland, both near the industrial area. Each profile was sampled in excavated pits up to a depth of 100 cm and loose randomly-oriented soil samples (~500 g) were taken every 2 cm along the upper 50 cm, and extracted each 5 cm along the deepest 50 cm. Dustfall samples were collected monthly from April 2012 to October 2017 (D201204–D201710, 64 samples). The sampling instrument, a custom-made cylinder 70 cm in height and 35 cm in diameter, was located in an open area and set up on the roof of a 3 m tall building near the smelting plant. 

### 2.2. Laboratory Measurements

All samples were air-dried in the laboratory, and topsoil samples sieved through a 0.9 mm mesh to remove all hair, plant debris, plastic residue and small stones before conducting the laboratory measurements. The magnetic measurements were performed according to this sequence: (i) low frequency (470 Hz) magnetic susceptibility (*χ*_lf_) and high frequency (4700 Hz) magnetic susceptibility (*χ*_hf_) were measured using a MS2B magnetometer (Bartington Instruments, Liverpool, UK) then the percentage of frequency-dependent susceptibility (*χ*_fd_%) was calculated; (ii) Anhysteretic remanent magnetization (ARM), which was developed using a demagnetizer (DTECH, Narragansett, Rhode Island state, USA) in a peak alternating field (AF) of 100 mT and direct current (DC) biasing field of 0.1 mT, and then the susceptibility of anhysteretic remanent magnetization (*χ*_ARM_) was calculated through dividing by the intensity of the DC field; (iii) Saturation isothermal remanent magnetization (SIRM), which was imparted at 1 T using an MMPM10 Impulse Magnetizer (Munich, Germany); (iv) S_−100 mT_, which was imparted at 100 mT in the opposite direction; (v) Magnetic hysteresis loops and thermomagnetic curves (J-T curve), were determined using a variable field translation balance (VFTB), and the heating/cooling cycles measured from 25 °C to 700 °C and back in a 110 mT magnetic field performed in air. 

## 3. Results

### 3.1. Magnetic Parameters and Their Temporal and Spatial Distribution

#### 3.1.1. Topsoil

**Figure 2:**
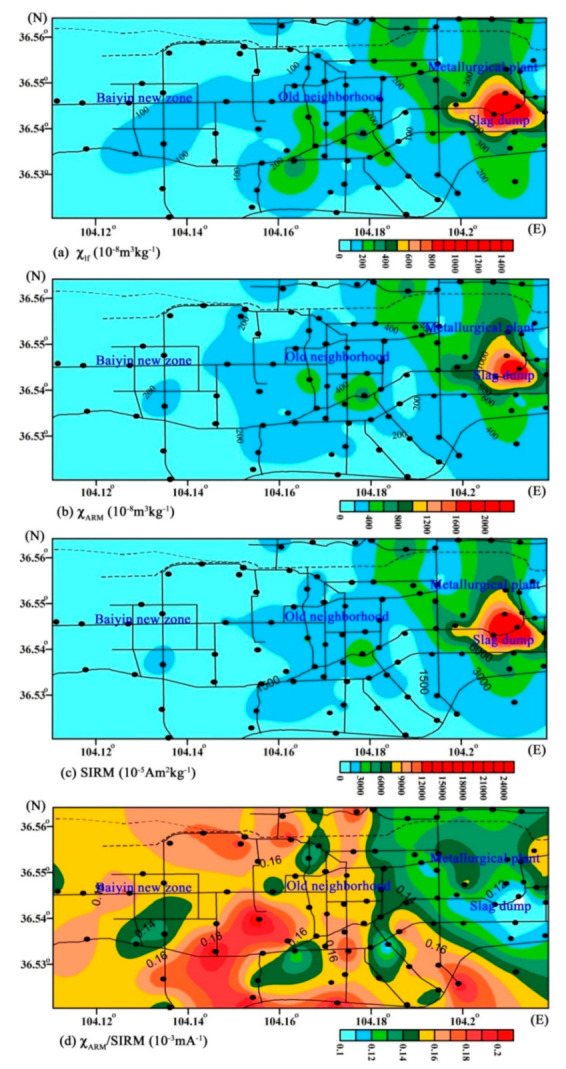
Isoline plot of magnetic parameters of χ_lf_, χ_ARM_, SIRM and χ_ARM_/SIRM for topsoil samples.

Table 1 reveals a wide range of *χ*_lf_ values, from 25 × 10^−8^ to 1485 × 10^−8^ m^3^ kg^−1^, with a mean value of 192 × 10^−8^ m^3^ kg^−1^ for soil samples. These values are greater than that from topsoils from megacities, e.g., Beijing [19], Shanghai [22], and Xi’an [23] (Table 2). The spatial distribution displays a distinct and large anomalous area adjacent to the metal plant and slag-heap, with *χ*_lf_ reaching values >300 × 10^−8^ m^3^ kg^−1^, while the *χ*_lf_ values decrease with increasing distance from the smelting plant. Minimum values of magnetic susceptibility occur in the western of Baiyin city, with values <100 × 10^−8^ m^3^ kg^−1^ (Figure 2a). The percentage of frequency-dependent susceptibility (χ_fd_%) values are below 3.49% for all samples, which indicates that the magnetic grains in the SP state do not contribute significantly to the susceptibility [24,25]. Generally, lower *χ*_ARM_/SIRM values are associated with coarse magnetite grains, while higher ratios represent finer grains, especially in the SD state. The *χ*_ARM_/SIRM values for all samples range from 0.10 × 10^−3^ mA^−1^ to 0.21 × 10^−3^ mA^−1^, with very low values near the slag heap (Figure 2d), confirming the predominant contribution of coarse magnetic particles in the most polluted area.

#### 3.1.2. Vertical Soil Profiles

**Figure 3:**
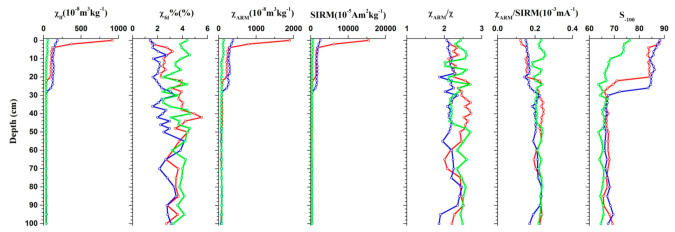
Magnetic parameters of vertical soil profiles: profile 1 was shown in green; profile 2 was shown in blue; profile 3 was shown in red.

Figure 3 illustrates the vertical variations of magnetic parameters at different depths. Profile 1 was located in the suburban areas with low vegetation cover and few signs of direct human activities. The soil core presents a low mean χ_lf_ value of 36 × 10^−8^ m^3^ kg^−1^, and displays a weak decreasing trend from the top to the base layers. In the industrial areas, the upper levels of the two soil profiles show higher χ_lf_ values, suggesting that industrial activity can be considered as the main source of magnetic minerals. The highest magnetic susceptibility values were measured in the upper 26 cm of profile 2, whereas profile 3 displayed a sharp reduction in magnetic susceptibility values from the surface layer (0–2 cm), and then exhibiting a continues decrease, albeit less markedly, from 4 to 20 cm, suggesting a clear contribution from anthropogenic-related ferrimagnetically enriched minerals in the surface layers of the abandoned plant and surrounding field. Magnetic susceptibilities of profiles 2 and 3 reached the value of ~30–40 × 10^−8^ m^3^ kg^−1^ in the lower part (30–100 cm), with S_−100_ down to 67, significantly lower than in the upper half (79 for profile 2 and 84 for profile 3), indicating that the lower half samples contain greater quantities of high coercivity anti-ferromagnetic minerals, e.g., hematite, and that this magnetic signature could be primarily related to the lithogenic and pedogenic background. 

#### 3.1.3. Atmospheric Dustfall

**Figure 4:**
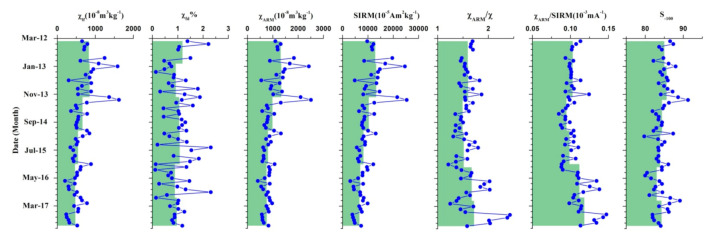
Monthly variations of magnetic parameters of atmospheric dustfall. The green part indicates the annual average concentration variation of each parameter.

Atmospheric dustfall samples present the greatest mean χ_lf_ value of 625 × 10^−8^ m^3^ kg^−1^ (Figure 4), which is significantly higher than that for cities much larger than Baiyin, such Beijing [14], Xi’an [32], Lanzhou [34] and Urumqi [29] (Table 2). Moreover, χ_lf_ values of dustfall in these large cities exhibit seasonal variations, with higher χ_lf_ values during winter (Dem - Feb) and lower values during summer and autumn, in association with coal combustion processes [34]. In the Baiyin study, highest χ_lf_ values are evident in the winter of 2012 and 2013, and slightly higher χ_lf_ values in winters of 2014, 2015 and 2016. In addition, the mean value of χ_lf_ presents clear annual variations and a decreasing trend (827 × 10^−8^ m^3^ kg^−1^, 848 × 10^−8^ m^3^ kg^−1^, 660 × 10^−8^ m^3^ kg^−1^, 543 × 10^−8^ m^3^ kg^−1^, 463 × 10^−8^ m^3^ kg^−1^, 472 × 10^−8^ m^3^ kg^−1^ for the years of 2012, 2013, 2014, 2015, 2016 and 2017, significantly), especially since 2014 (Figure 4). These results suggest that the contribution of industrial processes to atmospheric dustfall is falling with time. The ratio of S_−100_ enables the distinction of ferrimagnetic components from hard coercivity antiferromagnetic components. The higher values of S_−100_ (mean values = 84.34%) reflect the relatively high concentration of ferrimagnetic material (e.g., magnetite). 

### 3.2. Magnetic Mineralogy 

**Figure 5:**
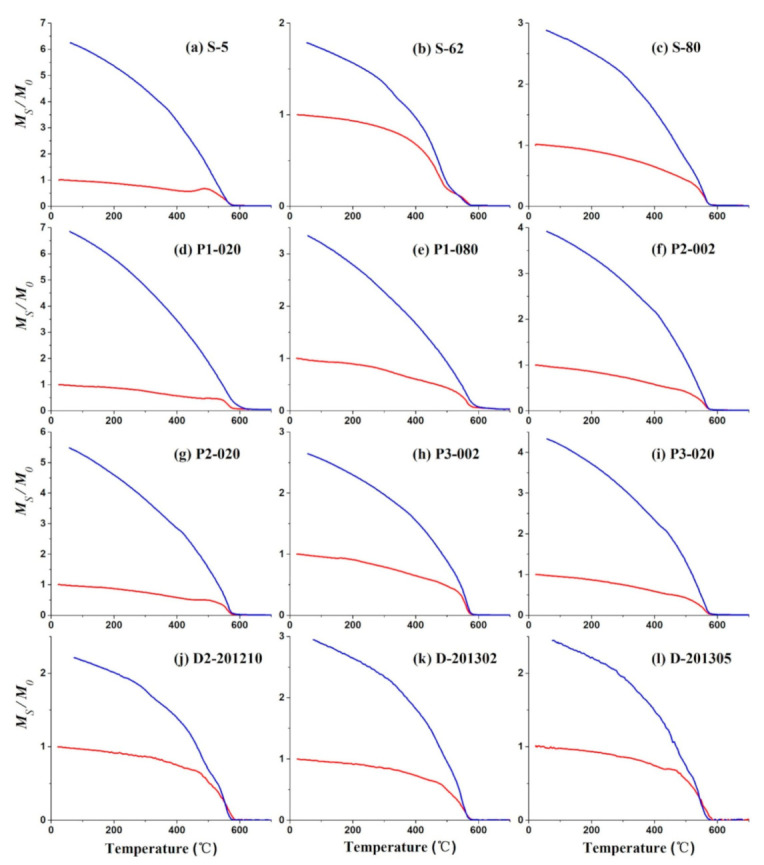
Thermomagnetic curves of typical soil samples. The red line is the heating curve, blue line is the cooling curve.

Magnetite is the main magnetic mineral in all samples, which is indicated by the Curie temperature (Tc) of ~580 °C displayed in the thermomagnetic curves (Figure 5) [38]. Samples around the slag dump have two thermomagnetic components, as illustrated by sample S-62, which revealed two Curie temperatures (Figure 5b). Together with the Curie temperature of magnetite (580 °C); another Tc is around 500 °C, which indicates impure magnetite in which Fe may be substituted by Al or Ti, maybe the Ti-poor titanomagnetite [39,40]. The thermomagnetic curves of the soil profile 1 showed two Tc of 580 °C and 680 °C, indicating that magnetite and hematite both dominate their magnetic properties (Figure 5d,e). 

**Figure 6:**
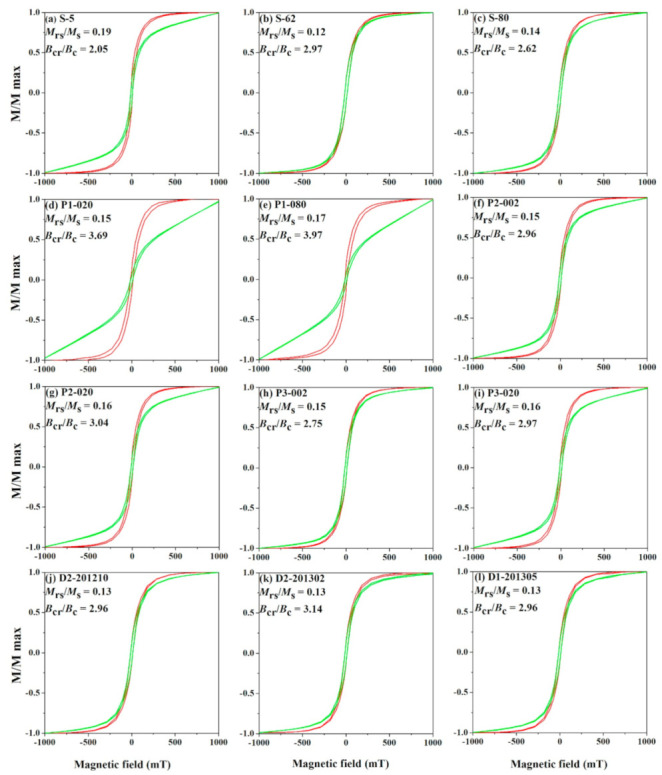
Magnetic hysteresis loops for representative samples following before (green line) and after (red line) subtraction of the paramagnetic contribution.

**Figure 7:**
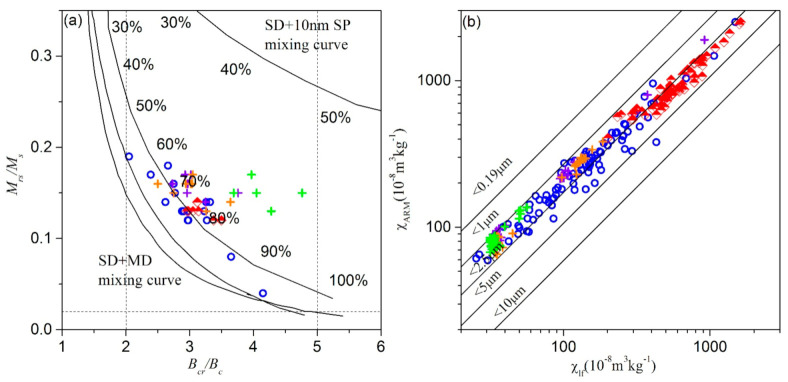
Scatter plots. (**a**) Day plot of the ratios M_rs_/M_s_ and B_cr_/B_c_. Single domain (SD), pseudo-single domain (PSD) and multi-domain (MD) ranges are shown after Dunlop [41]. (**b**) King plot between χ_lf_ and χ_ARM_ [42,43]. The blue hollow circle represents topsoil, the green cross represents soil profile 1, the orange cross represents soil profile 2, the purple cross represents soil profile 3, and the red square represents atmospheric dustfall.

The hysteresis loops of selected samples (Figure 6) show that most curves close below ~300 mT, indicating low coercivity ferromagnetic phases dominate the magnetic properties, while only soil profile 1 displays a high saturation field, ~500 mT, indicating the existence of paramagnetic minerals or high coercivity minerals (such as hematite) in background soil (Figure 6d,e). A Day-plot of hysteresis parameters of all samples shows that all samples locate within the pseudo-single domain (PSD) range [41] (Figure 7a), and the % multidomain (MD) contribution in soils and atmospheric dustfall samples around Cu-Pb-Zn smelter plant area is 70~80%, while it is higher (~90%) in samples subject to traffic pollution. The bivariate plots of χ_lf_ versus χ_ARM_ shows surface soil and atmospheric dustfall samples are in the particle size-range of 1.0–5 μm, while soil profile samples fall within 1.0–2.5 μm [42,43] (Figure 7b). This result suggests that magnetite grain size is finer in soil profiles.

In summary, evidence from thermomagnetic curves, hysteresis loops, Day plot and King plot all reveal that the particle size of magnetite is mainly located within the PSD range for all samples, whereas samples at 30–100 cm depth of the soil profile contain a slightly higher amount of SP particles and hematite. 

## 4. Discussion

### 4.1. Magnetic Properties of Different Environmental Carriers

There are many different potential causes of the enhancement of magnetic susceptibility, but they are generally related to (1) an increase in magnetic minerals due to pedogenesis; (2) anthropogenic factors, such as fossil fuel combustion, vehicle exhaust and industrial activities [44]. Previous studies have reported that the distribution of magnetic susceptibility values in soil profiles show higher concentrations of iron oxides in spodic (iron-rich) horizon at a depth between 30 and 40 cm for polluted forest soils, while the uppermost horizons displayed magnetic depletion [44,45,46]. Other studies report that highest susceptibility values are found in the upper 0–3 cm for forested areas [20,47] which is a similar pattern for soils in our study, where magnetic susceptibility values show strong magnetic enrichment in the uppermost horizons (0–2 cm), especially for the wasteland profiles. It should be noted that the phenomenon is not exhibited in the agricultural field. Due to long-term ploughing, the pollutants deposited on the surface soil are fully mixed with the original soil, so the magnetic susceptibility value of the 0–2 cm soil of the field surface is significantly lower than that of the wasteland. while the situation is opposite in the 2–30 cm layer, which suggests that farming practices such as ploughing transfer pollutants in the lower soil layers (below 3 cm), while areas free of ploughing display accumulation of dust mostly in thin layers at the surface of the soil. Therefore, reducing detrimental soil tillage practices can alleviate the vertical migration of resource-based urban pollution. 

**Figure 8:**
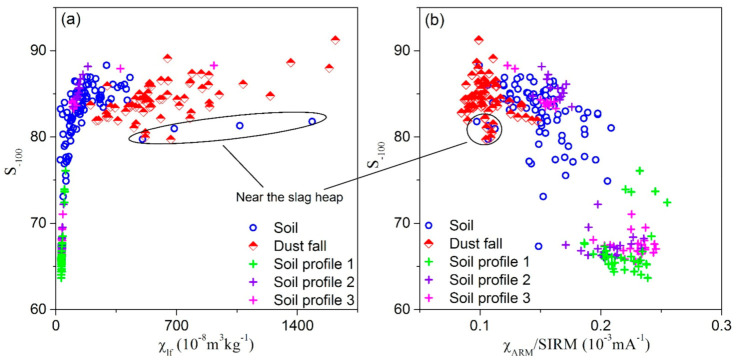
Scatter plot of (**a**) χ_lf_ versus S_−100_ and (**b**) χ_ARM_/SIRM versus S_−100_.

Urban particulate pollution is mainly a consequence the burning of coal and industrial processes in the cities of Northern China. In our study, the ferrimagnetic mineral content of dust samples during 2014 and 2017 were significantly lower than those in 2012 and 2013, which is suggested as relating to regional economic transformation. The mines of Baiyin City are today already exhausted, or nearly exhausted, after 50 to 60 years’ exploitation and the copper and lead-zinc smelter plant enterprises have been either discontinued or running at half capacity since 2014. Plotting S_−100_ against χ_lf_ and/or χ_ARM_/SIRM (Figure 8) further illustrates that the atmospheric dustfall plot cluster in a well-defined group, with increasing values of χ_lf_, low χ_ARM_/SIRM and relatively stable high S_−100_ values, indicating an important contribution from industrial emissions, such as fly ash derived from industrial combustion [16,48]. In contrast, surface soil samples gathered near the slag heap plot occupy a tightly clustered group exhibiting high χ_lf_, lower χ_ARM_/SIRM and lower S_−100_ values, suggesting relatively lower magnetite concentrations and finer ferrimagnetic grains compared with atmospheric dustfall. Therefore, the magnetic parameters of samples identify specific pollution signals for different environmental carriers, and reflect spatial and temporal changes in pollution sources in Baiyin city. 

### 4.2. Source Allocation of Different Environmental Carriers

**Figure 9:**
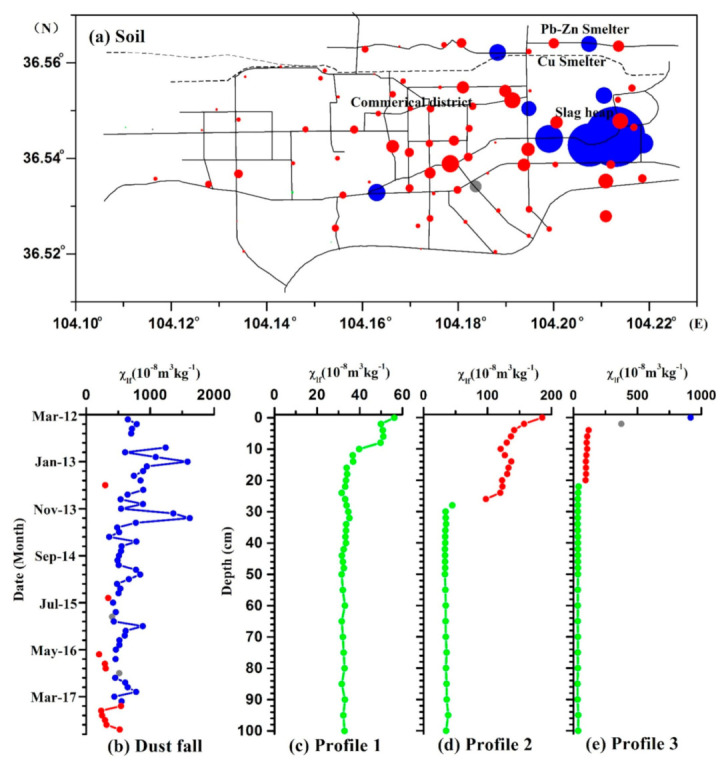
Spatial distribution of the degree of affinity of each sample to each of its possible (fuzzy) clusters. (**a**) topsoil; (**b**) atmospheric dustfall; (**c**) soil profile 1; (**d**) soil profile 2; (**e**) soil profile 3. Green dots represent samples of cluster 1, red dots samples of cluster 2, and blue dots samples of cluster 3.

Cluster analysis (fuzzy c-means cluster analysis) was carried out using four diagnostic magnetic parameters (χ_lf_, χ_ARM_/SIRM, HARD% and S_−100_) to identify smelting plant and motorized traffic emissions by magnetic fingerprinting. These parameters can be controlled by the mineralogy and/or the magnetic particles grain size. The fuzzy cluster analysis methods used here are described in detail in Wang [15] and Hansard [49]. When all samples, including surface soil, soil profile and atmospheric dustfall, are included in the fuzzy cluster algorithm, three distinct (and statistically optimal) groups are identified by their cluster affinities (Table 3) and sample locations (Figure 9).

Cluster 1 exhibits very low χ_lf_ and S_−100_ values, but high of χ_fd_% and χ_ARM_/SIRM values, reflecting low magnetite concentrations, higher antiferrimagnetic minerals content and ultra-fine magnetic particulates. Some surface soil samples collected predominantly within the western boundary of Baiyin city, remote from the principal polluting industries, and soil profile 1 together with the samples below the upper 26 cm in Profile 2 and 20 cm in Profile 3, belong to this cluster. Moreover, mineralogical analysis reveals that magnetite and hematite dominate the magnetic properties of these samples. These results reveal that the lithogenic and pedogenic background dominates the magnetic properties [50], thus the background magnetic susceptibility in Baiyin city can be considered as having a low value (37 × 10^−8^ m^3^ kg^−1^).

Cluster 2 displays higher magnetite concentrations and coarser magnetic grain sizes, and includes most surface soil samples from the commercial district and samples from the upper 26 cm in Profile 2 and upper 20 cm in Profile 3, dustfall samples collected in the summer of 2016 and 2017 belong to this cluster. The mean χ_lf_ value is 156 × 10^−8^ m^3^ kg^−1^, which is 4 times greater than that of the background. Thus, this cluster indicates moderate pollution. Our previous study showed that traffic pollution and industrial dust sources were dominant in soil samples from the commercial district of Baiyin city [15]. Soil profiles 2 and 3 were located near the smelter plants, therefore the upper soil samples were affected by plant emissions from this source and by the transport of surface pollutants. Cluster 2, therefore, suggests a source combining traffic and industrial pollution.

Cluster 3 has significantly higher values of χ_lf_ and S_−100_ and significantly lower values of χ_fd_% and χ_ARM_/SIRM relative to that of clusters 1 and 2. The magnetic susceptibility is 663 × 10^−8^ m^3^ kg^−1^, which is 18 times greater than the background, indicating that these samples are strongly polluted. Cluster 3 contains surface soil samples collected near the smelting plant and atmospheric dustfall with high magnetite content and coarser magnetic particle sizes. Such magnetic properties might primarily be related to industrial processes, such as emissions from factory chimneys and blowing dust from slag heaps and mineral transport. However, thermomagnetic curves indicate that soil samples near the smelting slag contain some impure magnetite. In our study, there are slag heaps and a tailings dam near the smelting plant, which constitute a potential pollution source. Atmospheric dustfall mainly comes from the combination of local emissions and pollutants transported from surrounding counties. Moreover, the dustfall sampling points were located downwind of the smelting plant, therefore fly ash diverted from the plant’s chimneys is the major contributor of atmospheric dustfall. The result also implies that the mineral characteristics of dustfall derived from smelting slag and smokestacks are significantly different.

## 5. Conclusions

PSD magnetite dominates the magnetic properties of soils and atmospheric dustfall in Baiyin City with a background magnetic susceptibility of 37 × 10^−8^ m^3^ kg^−1^. The spatial distribution of soil samples reveals low magnetite concentrations in the western area of the city, with higher contents in the industrial area of northeastern Baiyin, indicating that its primary source is the smelting plant. The vertical distribution of magnetic minerals in soils confirms that the top 0–20 cm are enriched in pollutants suggesting that planting crops will accelerate the transfer of contaminants deeper in the soil; therefore, reducing soil tillage practices can mitigate the vertical migration of resource-based urban pollutants.

Seasonal variations are evident in the atmospheric dustfall measurements, suggesting that industrial emissions by factory chimneys and blowing dust from slag heaps and mineral transport control its magnetic properties, and that, furthermore, coal burning in winter is not the dominate source. Since the smelter plant ceased to operate at full capacity since 2014, the dominant source of atmospheric dustfall changed from fly ash emissions by factory chimneys and mineral dust to blowing dust from slag heaps. Governance of slag pollution is then a major task in resource-exhausted urban areas. In conclusion, combined magnetic measurements of multiple environmental carriers, such as soil and atmospheric dustfall, provide accurate, integrated, and detailed pollution proxies to highlight temporal and spatial scale variations in pollution sources.

## Figures and Tables

**Table 1:** Summary of the magnetic parameters of topsoil, soil profiles and atmospheric dustfall from Baiyin city.

		χ_lf_ (10^−8^ m^3^ kg^−1^)	χ_fd_% (%)	χ_ARM_ (10^−8^ m^3^ kg^−1^)	SIRM (10^−5^ Am^2^ kg^−1^)	χ_ARM_/ χ	χ_ARM_/SIRM (10^−3^ mA^−1^)	S_−100_
Soil	Mean	192	1.88	336	2541	1.88	0.15	82.74
	Min-Max	25–1485	0.53–3.49	60–2509	313–25,791	0.89–2.70	0.10–0.21	67.34–88.31
	sd	207	0.66	329	3259	0.34	0.02	3.52
	CV	1.08	0.35	0.98	1.28	0.18	0.16	0.04
Profile 1	Mean	36	3.70	85	386.22	2.37	0.22	66.85
	Min-Max	32–56	2.42–4.61	68–137	332–588	2.02–2.69	0.19–0.25	63.67–76.09
	sd	7	0.66	20	72	0.20	0.02	3.39
	CV	0.20	0.18	0.23	0.19	0.08	0.08	0.05
Profile 2	Mean	73	2.47	158	912	2.16	0.19	74.36
	Min-Max	33–185	1.42–4.15	65–383	351–2538	1.86–2.47	0.14–0.24	66.06–88.17
	sd	52	0.71	110	739	0.15	0.03	9.12
	CV	0.71	0.29	0.70	0.81	0.07	0.14	0.12
Profile 3	Mean	86	3.34	192	1232	2.39	0.20	72.62
	Min-Max	32–916	1.41–5.53	68–1896	345–15,454	2.01–2.71	0.12–0.25	65.57–88.29
	sd	200	0.96	411	3421	0.21	0.04	8.34
	CV	2.34	0.29	2.14	2.78	0.09	0.19	0.11
Atmospheric dustfall	Mean	625	1.00	1140	9538	1.88	0.11	84.34
	Min-Max	201–1619	0.11–2.30	407–2504	3027–25,308	1.21–2.42	0.08–0.15	79.72–91.23
	sd	313	0.54	459	4923	0.26	0.01	2.34
	CV	0.50	0.54	0.40	0.52	0.14	0.14	0.03

**Table 2:** Variations in population density and magnetic parameters of various environmental carriers in different cities in China.

City Name	Type	Area of Built Districts/km^2^ [26]	Population Density (Persons/km^2^) [26]	Number of Samples	χ_lf_ (10^−8^ m^3^ kg^−1^)	SIRM (10^−5^ Am^2^ kg^−1^)	χ_fd_% (%)	Reference
Beijing	Soil	1261	1006	63	179	1967	2.25	[19]
	Street dust			63	433	4517	1.28	[19]
	Dustfall			24	209	6432		[14]
Shanghai	Soil	886	2635	97	176	2421	2.47	[22]
	Street dust			439	825	10,148	3.47	[27]
Hangzhou	Soil	453	1452	182	128	2562	3.60	[28]
Urumqi	Soil	384	263	85	281	2895	1.80	[21]
	Dustfall			12	509	2095	2.17	[29]
Xi’an	Soil	375	1599	34	182		5.89	[23]
	Street dust			97	530	6366	4.29	[30]
	Dustfall			24	357	5058	2.26	[31]
Xuzhou	Soil	274	1056	167	234	2435	2.78	[32]
Lanzhou	Soil	199	1265	117	219	2508	2.20	[33]
	Street dust			71	450	6618	2.22	[17]
	Dustfall			47	544	9555	1.80	[34]
Luoyang	Soil	187	3612	215	236		4.32	[35]
Yinchuan	Soil	135	434	75	117	1519	2.00	[36]
Baiyin	Soil	58	141	87	192	2540	1.88	This paper
	Street dust			43	246	3768	1.22	[37]
	Dustfall			64	625	9538	1.00	This paper

**Table 3:** Parameter means for the four-cluster solution.

	χ_lf_ (10^−8^ m^3^ kg^−1^)	χ_fd_% (%)	χ_ARM_/SIRM (10^−3^ mA^−1^)	S_−100_
Cluster 1	37	3.48	0.22	67.50
Cluster 2	156	1.91	0.15	83.42
Cluster 3	663	0.96	0.10	84.28

## Data Availability

All relevant data sets in this study are described in the manuscript.
